# PREVENTT: preoperative intravenous iron to treat anaemia in major surgery: study protocol for a randomised controlled trial

**DOI:** 10.1186/s13063-015-0774-2

**Published:** 2015-06-04

**Authors:** Toby Richards, Ben Clevenger, Jane Keidan, Tim Collier, Andrew A. Klein, Stefan D. Anker, John D. Kelly

**Affiliations:** Division of Surgery and Interventional Science, University College London, London, UK; Consultant Haematologist (retired), Queen Elizabeth Hospital, King’s Lynn NHS Trust, Suffolk, UK; Department of Medical Statistics, London School of Hygiene and Tropical Medicine, London, UK; Department of Anaesthesia and Intensive Care, Papworth Hospital, Cambridge, UK; Department of Innovative Clinical Trials, University Medical Centre Göttingen (UMG), Göttingen, Germany; Division of Surgery and Interventional Science, 4th Floor, UCL Medical School Building, 21 University Street, University College London, London, WC1E 6AU UK

**Keywords:** Anaemia, Intravenous iron, Surgery, Clinical trial, Blood transfusion, Patient blood management

## Abstract

**Background:**

Anaemia is common in patients undergoing major surgery. The current standard of care for patients with low haemoglobin in the peri-operative period is blood transfusion. The presence of preoperative anaemia is associated with an increased likelihood of the patient receiving peri-operative transfusion and worsened outcomes following surgery, more post-operative complications, delayed recovery and greater length of hospital stay. Intravenous iron, if applied in the preoperative setting, may correct anaemia by the time of surgery and reduce the need for blood transfusion and improve outcomes.

**Methods/Design:**

PREVENTT is a phase III double-blind randomised controlled trial that will compare the use of intravenous ferric carboxymaltose (dose 1000 mg) with placebo 10–42 days before major open abdominal surgery in 500 patients with anaemia (haemoglobin < 120 g/L). The primary outcome measure will be the need for blood transfusion and secondary endpoints will include post-operative recovery, length of hospital stay, health care utilisation and cost analysis.

**Trial registration:**

ISRCTN67322816 – registered 9 October 2012.

ClinicalTrials.gov identifier: NCT01692418.

## Background

The World Health Organisation defines anaemia as insufficient red blood cell (RBC) mass to meet the body’s physiological needs with a haemoglobin (Hb) concentration of < 130 g/L for men and < 120 g/L for non-pregnant women [1]. Anaemia is associated with impaired physical function, reduced quality of life, infection, increased patient morbidity and mortality [2]. Preoperative anaemia is common, affecting 30–60 % of all patients undergoing major elective surgery [3]. In the surgical setting anaemia compounds the stress of operation; anaemia is an independent risk factor for blood transfusion, in-patient complications, delayed hospital discharge and poorer recovery [4]. The causes for anaemia in this patient group are often multifactorial and can be due to blood losses, nutritional causes, functional iron deficiency associated with elevated hepcidin (cancer and/or inflammatory disease – anaemia of chronic disease (ACD)), or a combination of these [5]. Two main types of anaemia affect surgical patients, iron deficiency anaemia (IDA) and ACD; the latter is more common in chronically ill and hospitalised patients [6]. ACD can be difficult to diagnose, often being regarded as a diagnosis of exclusion. A key feature of ACD is a disruption of normal iron homeostasis initiated by a cytokine-mediated immune response, such as in chronic inflammatory disease, during infection or following surgery [6, 7]. This inability to define ‘iron deficiency’ in patients with anaemia before operation has meant that most patients are not treated with iron therapy, and blood transfusion in the peri-operative period remains the standard of care [8].

The demand for blood components increases every year. In 2008 to 2009 1.86 million units of blood were transfused in the UK at an overall cost of provision to the NHS of £247.4 million. Although anaemia increases the requirement for transfusion, blood transfusion itself has been associated independently with a worse patient outcome. Prospective observational studies suggest that allogenic blood transfusion (ABT) increases the risk of post-operative complications and longer hospital stay [9, 10].

Intravenous (IV) iron is the standard of care for iron supplementation in patients with iron deficiency anaemia in chronic renal failure [11]. Its use has widened to routinely treat anaemia in patients with inflammatory bowel disease [12], and cardiac disease [13]. The introduction of new IV iron preparations that can be administered as a single treatment of up to 1000 mg in a relatively short (15 min) time without the need for a test dose and with low risk of reactions, have facilitated small trials within the fields of obstetric, gynaecological and orthopaedic surgery. These studies have suggested that IV iron may rapidly increase Hb levels before operation and this may result in lower transfusion rates [14–20]. Two recent reviews called for a randomised controlled trial (RCT) on the role of IV iron in surgery to prevent blood transfusion [21, 22].

We aim to assess whether a single treatment dose of IV ferric carboxymaltose (FCM) given to anaemic patients prior to major abdominal surgery would reduce the need for blood transfusion in the peri-operative period. Furthermore, this intervention may reduce post- operative complications and overall prove cost-effective.

## Methods/Design

Adult patients undergoing elective major open abdominal surgery, defined as an operation of anticipated duration more than 1 h, will be eligible for screening. The indication for operation may be for benign or malignant disease.

To date, multiple studies have compared laparoscopic to open surgery. Laparoscopic surgery is now routine practice in many areas of surgery. Cochrane database reviews suggest a benefit in reducing the need for blood transfusion and reduced length of hospital stay [23–25]. In major abdominal surgery such as gastrectomy, colectomy, nephrectomy or cystectomy and hysterectomy (for malignant disease) open surgery remains the mainstay of treatment. In the majority of open cases patients have complex disease or require extensive surgery. Consequently these patients have a higher event rate. In contrast, patients undergoing laparoscopic surgery may be more selected; often for benign disease or in cases where the surgery may be less extensive or technically easier. In laparoscopic surgery the post-operative event rate may be lower. Therefore, it was decided to exclude laparoscopic surgery because of a need to increase the overall number of participants in the study to allow detection of a difference in endpoints.

Patients will be screened for inclusion into the trial at a normal routine hospital attendance, such as outpatient clinic visits or attendance for tests as part of their evaluation for surgery (for example radiological tests or endoscopy). Those with screening Hb 90–120 g/L and able to receive the study infusion 10 days to 42 days before planned operation, will be eligible for randomisation. Prior to inclusion, freely given written informed consent must be obtained from all patients. Exclusion criteria include patients undergoing laparoscopic surgery; body weight under 50 kg; known history of acquired iron overload; family history of haemochromatosis or thalassemia or transferrin saturation (TSAT) > 50 %; known reason for anaemia (e.g. untreated vitamin B_12_ or folate deficiency or haemoglobinopathy); treatment with erythropoietin, IV iron therapy or blood transfusion in the previous 12 weeks; known hypersensitivity to FCM or its excipients; temperature > 37.5 °C or receiving non-prophylactic antibiotics; chronic liver disease and/or screening alanine transaminase (ALT) or aspartate transaminase (AST) above 3 times the upper limit of the normal range and those with severe asthma or severe allergy.

The study protocol was approved by the National Research Ethics Committee East of England – Welwyn, 5 November 2012, reference 12/EE/0445 EudraCt 2012-002786-35. Further, the trial was registered in the US clinical trials database NCT01692418.

### Patient outcomes measures

The co-primary outcomes to be investigated are the risk of blood transfusion (receiving any volume of 1 unit or more than 1 unit of packed red cells or any other blood component) or death, from randomisation until 30 days following the index operation, and the blood transfusion rate (including repeat transfusions) from randomisation until 30 days following the index operation. Where more than 1 unit of packed red cells or any other blood component is intended to be received contiguously this is regarded as a single blood transfusion (including single unit transfusion practice when units are given contiguously). The blood transfusion rate is defined as the number of blood transfusions divided by the total patient time at risk.

Secondary outcome measures include the change in haemoglobin levels from randomisation to day of index operation, 8 weeks post index operation and 6 months post index operation; total number of units of blood or blood components cross matched; total number of packed red cells and any blood components transfused from randomisation to 30 days post index operation; post-operative complications and the Post Operative Morbidity Survey (POMS) outcome at days 3, 5, 7 and 14 following the index operation [26].

Health-related Quality of Life (HRQoL) outcomes to be assessed are change in the Multidimensional Fatigue Inventory (MFI) questionnaire total score and European Quality of Life: 5 Dimensions-5 Levels (EQ-5D-5 L) questionnaire total score from baseline at day 10–8 weeks and 6 months post-operatively and change in Single Question Outcome Measure (SQOM).

Health economics assessments will be health care resource utilisation from baseline to 6 months post surgery; calculated NHS and societal costs from baseline to 6 months post surgery; Quality-adjusted Life Years (QALYs) from baseline to 6 months post surgery; cost-effectiveness measured in terms of the incremental cost per percent reduction in patients receiving blood transfusions; and incremental QALYs gained, using data from baseline to 6 months post surgery.

**Fig. 1:**
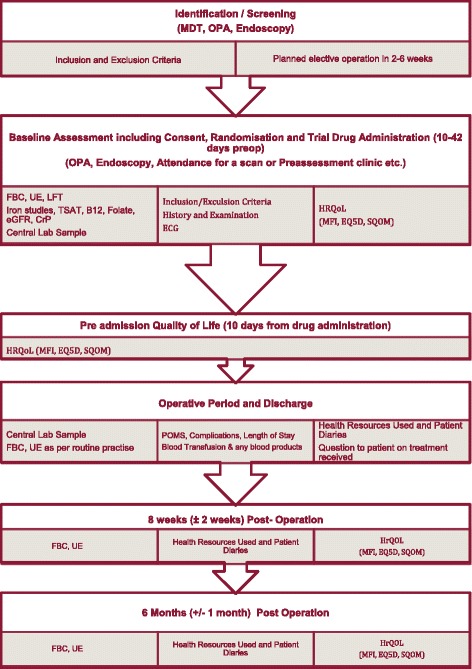
Assessments flow diagram

The following safety and related efficacy outcomes will be measured: any reaction or side effect from trial therapy; any reaction or side effect from whole blood or blood component, transfusion reaction; serious adverse events (SAE) and suspected unexpected serious adverse reactions (SUSARs); length of hospital stay; mortality at 8 weeks and at 6 months post-operatively; readmission to hospital for any reason within 8 weeks and within 6 months of the index operation; rate of recurrent hospitalisations for any reason; death; blood transfusion from randomisation to 8 weeks and to 6 months post-operatively; change in estimated glomerular filtration rate (eGFR) during follow-up; changes in vital signs and laboratory test data. See Fig. 1 for Assessments flow diagram.

### Assessments

The ‘baseline assessments’, including laboratory tests for the purposes of the PREVENTT trial, are the same as those for routine clinical care in pre-assessment before major surgery. The screening and baseline period will not exceed 4 weeks, including laboratory tests taken within 4 weeks (full blood count (FBC), creatinine, urea and electrolytes (UE), liver function tests (LFT), iron studies, estimated glomerular filtration rate eGFR, C-reactive protein (CRP), thyroid function tests (TFT), vitamin B_12_ and folate); documentation of past medical history, vital signs (blood pressure, pulse rate, body weight, height, temperature. Additional blood samples will be taken for central laboratory analysis (FBC, iron studies, TSAT, total iron binding capacity (TIBC)). This way the site will remain blinded to any change in Hb post randomisation.

### Randomisation and blinding

Patients will be allocated to active treatment (FCM) or placebo in a 1:1 ratio using minimisation (with a random element incorporated) taking into account age (<70 years/70+ years), baseline haemoglobin (<100/100+ g/L), centre and type of operation (major, major-plus or complex).

### Blinding

FCM solution is dark brown in appearance. Blinding will be achieved by shielding the patients from seeing preparation of the study drug and having unblinded study personnel not involved in any study assessments (efficacy or safety) responsible for preparing and administering the study treatment. Preparing and administering the study drug behind a screen or curtain will achieve this. The drug will then be shielded from vision (Opabag (B Braun, Melsungen, Germany)) light protection bags) and administered through black tubing (Intrafix Air P with black pipe (B Braun, Melsungen, Germany)).

### Sample size and justification

The sample size requirement was calculated for the composite primary endpoint of blood transfusion or death by 30 days. The assumptions for the sample size calculations were based on data from the pilot study and observational trials and audits. The anticipated risk of blood transfusion in the control group is approximately 40 %. Using a type-1 error rate of 5 % and allowing for a 5 % loss in follow-up, recruiting 500 patients (250 in each group) will give 90 % power to detect an absolute reduction in risk of 14 % (i.e. 35 % relative risk reduction, RR = 0.65) in the treatment group and it will give approximately 80 % power to detect an absolute risk reduction of 12 % (i.e. 30 % RR reduction)).

## Discussion

The problem of anaemia in patients undergoing major surgery is being increasingly recognised [4]. No major clinical trial has been conducted to address the correction of iron deficiency and anaemia by the use of parenteral iron in patients undergoing major surgery. The NHS Enhanced Recovery Partnership Programme (ERP) [27] has highlighted the need to address anaemia in surgical patients as a correctable condition, but no specific recommendations or guidelines for the evaluation or treatment of anaemia in these patients have been proposed.

PREVENTT is designed as a randomised, double-blind, parallel-group, placebo-controlled, multi-centre study to investigate the efficacy and safety of IV iron compared to that of placebo in a patient population with anaemia undergoing major surgery. The dosing is based on the maximum dose of IV FCM (Ferinject®, Vifor Pharma UK, Bagshot, UK) that can be safely given in a reasonable time period.

As anaemia treatment is not standard care in these patients it is deemed acceptable to use a placebo control group for the supplemental IV iron therapy. The trial methodology is based on the FAIR-HF trial [28]. In recent trials in patients with chronic heart failure who demonstrate iron deficiency with and without anaemia, these were randomised to receive FCM or placebo for 6–12 months [13, 29].

The trial design is pragmatic and aims to include the proposed intervention within current NHS timelines. Standard practice is for patients to attend the surgical pre-assessment clinic 10 days to 6 weeks prior to operation. This is the same time period required for IV iron to produce an effective rise in Hb levels. Therefore, anticipated surgery in 10 days to 6 weeks was defined in the inclusion criteria.

The paradigm of patient blood management is becoming increasingly accepted in order to reduce unnecessary blood transfusions and to improve outcomes after surgery. It is defined as the timely application of evidence-based medical and surgical concepts designed to maintain haemoglobin concentration, optimise haemostasis and minimise blood loss in an effort to improve patient outcome [30]. It consists of 3 pillars: 1) the optimisation of erythropoiesis; 2) minimising blood loss and bleeding and 3) harnessing patient reserve and the tolerance of anaemia. The recognition and treatment of anaemia in the preoperative period is key to the first pillar. This must be integrated with other interventions, including preventing coagulopathy and bleeding and increased usage of restrictive transfusion thresholds [31, 32], in a multimodal, multidisciplinary approach to improve patient outcomes.

## Trial status

The study opened to recruitment in September 2013 and is anticipated to close in August 2016.

## Abbreviations

ABT: allogenic blood transfusion
ACD: anaemia of chronic disease
ALT: alanine transaminase
AST: aspartate transaminase
CRP: C-reactive protein
eGFR: estimated glomerular filtration rate
EQ-5D-5 L: European Quality of Life: 5 Dimensions-5 Levels
ERP: Enhanced Recovery Partnership Programme
FBC: full blood count
FCM: ferric carboxymaltose
Hb: haemoglobin
HRQoL: Health-related Quality of Life
IDA: iron deficiency anaemia
IV: intravenous
LFT: liver function tests
MFI: Multidimensional Fatigue Inventory
NHS: National Health Service
POMS: Post Operative Morbidity Survey
QALY: Quality-adjusted Life Year
RBC: red blood cell
RCT: randomised controlled trial
RR: relative risk
SAE: serious adverse events
SQOM: Single Question Outcome Measure
SUSAR: suspected unexpected serious adverse reactions
TFT: thyroid function tests
TIBC: total iron binding capacity
TSAT: transferrin saturation
UE: urea and electrolytes
