# Erratum to: PREVENTT: preoperative intravenous iron to treat anaemia in major surgery: study protocol for a randomised controlled trial

**DOI:** 10.1186/s13063-015-0869-9

**Published:** 2015-07-24

**Authors:** Toby Richards, Ben Clevenger, Jane Keidan, Tim Collier, Andrew A. Klein, Stefan D. Anker, John D. Kelly

**Affiliations:** Division of Surgery and Interventional Science, University College London, London, UK; Queen Elizabeth Hospital, King’s Lynn NHS Trust, Suffolk, UK; Department of Medical Statistics, London School of Hygiene and Tropical Medicine, London, UK; Department of Anaesthesia and Intensive Care, Papworth Hospital, Cambridge, UK; Department of Innovative Clinical Trials, University Medical Centre Göttingen (UMG), Göttingen, Germany; Division of Surgery and Interventional Science, 4th Floor, University College London, UCL Medical School Building, 21 University Street, London, WC1E 6AU, UK

After publication of this work [1], it has come to our attention that the following statement was missing from the acknowledgements section:

“The views and opinions expressed therein are those of the authors and do not necessarily reflect those of the HTA, NIHR, NHS or the Department of Health.”

The full acknowledgements section can be found below:

This project was funded by the National Institute for Health Research Health Technology Assessment (NIHR HTA) Programme (project number 10/104/06). Thank you to the trial coordinating staff at the LSHTM CTU. The views and opinions expressed therein are those of the authors and do not necessarily reflect those of the HTA, NIHR, NHS or the Department of Health.

## References

[1] Richards T, Clevenger B, Keidan J, Collier T, Klein AA, Anker SD (2015). PREVENTT: preoperative intravenous iron to treat anaemia in major surgery: study protocol for a randomised controlled trial. Trials.

